# Management of Oral Mucosal Fragility: A Pediatric Dental Perspective on a Unique Case of Epidermolysis Bullosa Simplex (EBS)

**DOI:** 10.7759/cureus.104056

**Published:** 2026-02-22

**Authors:** Aman Singh, Nidhi Chaurasia, Meenakshi Chandel, Vinay Srivastava, Sheetal Badnaware

**Affiliations:** 1 Pedodontics and Preventive Dentistry, Faculty of Dental Sciences, Institute of Medical Sciences, Banaras Hindu University, Varanasi, IND

**Keywords:** epidermolysis bullosa simplex (ebs), mucosal fragility, oral hygiene, pedodontic intervention, periodontal deterioration

## Abstract

Oral healthcare is severely hampered in patients with epidermolysis bullosa simplex (EBS), a rare inherited genodermatosis marked by mucocutaneous fragility and blister formation. This case report describes the dental management of a six-year-old female child clinically diagnosed with EBS presenting with severe periodontal manifestations such as extensive calculus accumulation, gingival bleeding, halitosis, and poor oral hygiene as a result of frequent oral ulcerations. Meticulous behaviour modification technique and atraumatic periodontal intervention were employed to minimize soft tissue injury. This report highlights the importance of early dental referral, preventive-focused care, and a multidisciplinary approach in managing pediatric patients with EBS.

## Introduction

Epidermolysis bullosa (EB) is a rare genetic dermatological disorder that is characterized by blister formation in the skin and mucous membranes. This is usually due to tissue separation, which occurs at variable depths, mainly due to an increased fragility of the skin [1]. This dermal condition has an estimated incidence of 1:50,000 live births in the USA, whereas the prevalence of this condition is estimated to be around 1:20,000-1:100,000 in Europe [2]. Indians have an estimated incidence of 54 per million live births for this inherited dermal condition [3]. This condition is usually caused by genetic variations in the proteins encoding for the dermal-epidermal adhesion area in the skin and mucous membrane. EB presents clinically as blisters and erosions on the affected areas, which vary in size and depth depending on the clinical phenotype of the condition. These eruptions on skin and mucous membrane may occur spontaneously or due to friction while rubbing, or they may also occur inadvertently in response to minor trauma over those surfaces [4]. Utilising an “onion-skin approach”, the 2020 Consensus Classification for Epidermolysis Bullosa has reclassified this condition into four clinical phenotypes depending on the skin and mucosal level at which blister formation occurs: simplex-intraepidermal level(EBS), junctional-junctional level(JEB), dystrophic-dermal level (DEB), and Kindler-mixed level (KEB) [5]. Apart from cutaneous involvement, EB has significant oral complications. The fragile oral mucosa predisposes patients to recurrent blistering, ulcerations, pain, and secondary infections, which in turn compromise routine oral hygiene practices and increase the risk of plaque accumulation, gingivitis, periodontal inflammation, and dental caries. As a result, pediatric dental management requires meticulous planning, atraumatic techniques, and preventive-focused care.

The current case report describes a case of a six-year-old female pediatric patient with epidermolysis bullosa simplex (EBS) diagnosed clinically, who was then referred for dental management. The present case is significant because it highlights severe periodontal deterioration secondary to avoidance of oral hygiene practices in a young child with clinically diagnosed EBS, an oral presentation that is less frequently emphasized compared to enamel defects or caries patterns reported in other EB subtypes. In addition, this case emphasizes the importance of behavioral, preventive, and minimally traumatic periodontal management strategies required in pediatric patients with mucosal fragility and thus reinforces the critical role of early dental referral in preventing avoidable oral morbidity.

## Case presentation

A six-year-old female pediatric patient, born to a non-consanguineous marriage and prematurely at preterm (28 weeks), with a birth weight of 2 kg, reported with the chief complaint of multiple, frequent ulcerations and pigmentations all over the body, and after adequate management, was referred from the Department of Dermatology for dental evaluation. The child had been clinically diagnosed with EBS by the Department of Dermatology based on characteristic mucocutaneous blistering precipitated by minor trauma and the absence of scarring or milia formation. Skin biopsy or genetic analysis had not been performed at the time of presentation due to financial constraints. There was no history of esophageal strictures, dysphagia, anemia, or growth retardation at the time of evaluation. The patient was under regular dermatological follow-up and was receiving supportive management, including topical corticosteroids and antibiotic preparations for secondary infections. The child was not able to brush properly and perform other oral hygiene practices for the past few years due to multiple and frequent ulcer formation intraorally. The parents also reported frequent bleeding from the upper and lower gums for the past six to seven months, which usually occurs on its own without any oral stimulation. The parents had consulted multiple dentists locally, where she was prescribed oral mouthwashes, but due to severe burning sensation on their consumption, the child had developed a fear of consuming any medicament orally and thus refrained from any oral hygiene practice further. The child’s behavior was definitely negative (a rating of 4 in the Frankl behaviour rating scale, Frankl et al. 1962) initially on the day of clinical presentation and was very much reluctant to open her mouth and behave properly in the dental operatory [6].

Extraoral examination

Face

**Figure 1:**
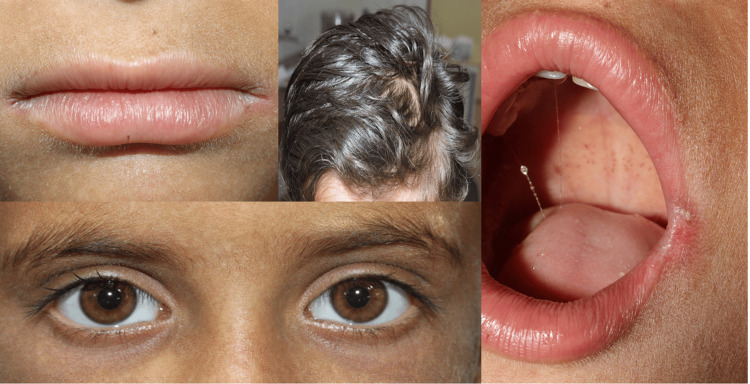
Clinical extraoral profile of a six-year-old epidermolysis bullosa simplex (EBS) child manifesting eyes (sclera), lips, hairs, and oral mucosa. The perioral region shows xerosis with mild fissuring and post-inflammatory changes of the lips consistent with skin fragility, scalp demonstrating areas of alopecia and sparse hair (secondary to recurrent blistering and healing, intraoral view showing erythematous patches on soft palate with stringy saliva without any obvious mucosal involvement and frank blistering visible and periocular region showing normal sclera and conjunctiva, highlighting the absence of ocular involvement in this case.

The facial skin displayed diffuse xerosis (dryness) with fine scaling and an overall rough texture. The perioral and periorbital areas had darker pigmentation and mild lichenification, suggesting chronic friction or dryness. There were no obvious erosions or crusting on the lips and oral commissures, but there was the presence of bilateral angular chelitis (mild form), suggesting chronic deficiency (Figure 1).

Lower Extremities

Both lower extremities demonstrated diffuse xerosis and hyperpigmentation with accentuated skin markings, and there were multiple patches of diffuse hyperkeratosis. Localized erosions were seen on the legs.

Feet and Nails

**Figure 2:**
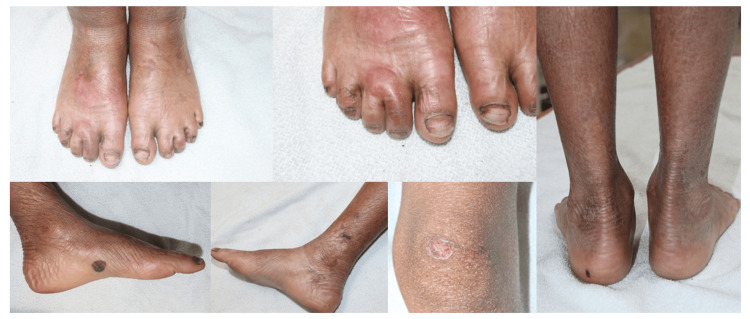
Extraoral manifestations on the lower limbs showing superficial bullae and erosions involving the feet.

The dorsal aspect of both feet revealed thickened skin with scaling and evidence of prior trauma. Several toenails showed dystrophy along with partial loss of nail plates and discoloration. There was hemorrhagic crusting and an exposed nail bed visible in some toes. On the lateral aspect of the foot, there was a well-defined dark hemorrhagic crust or possible blood-filled blister. There were scattered excoriated papules and hyperpigmented macules in the background of xerotic skin (Figure 2). The predominance of superficial trauma-induced blistering without significant scarring or pseudosyndactyly was consistent with the clinically suspected diagnosis of EBS.

Hands

**Figure 3:**
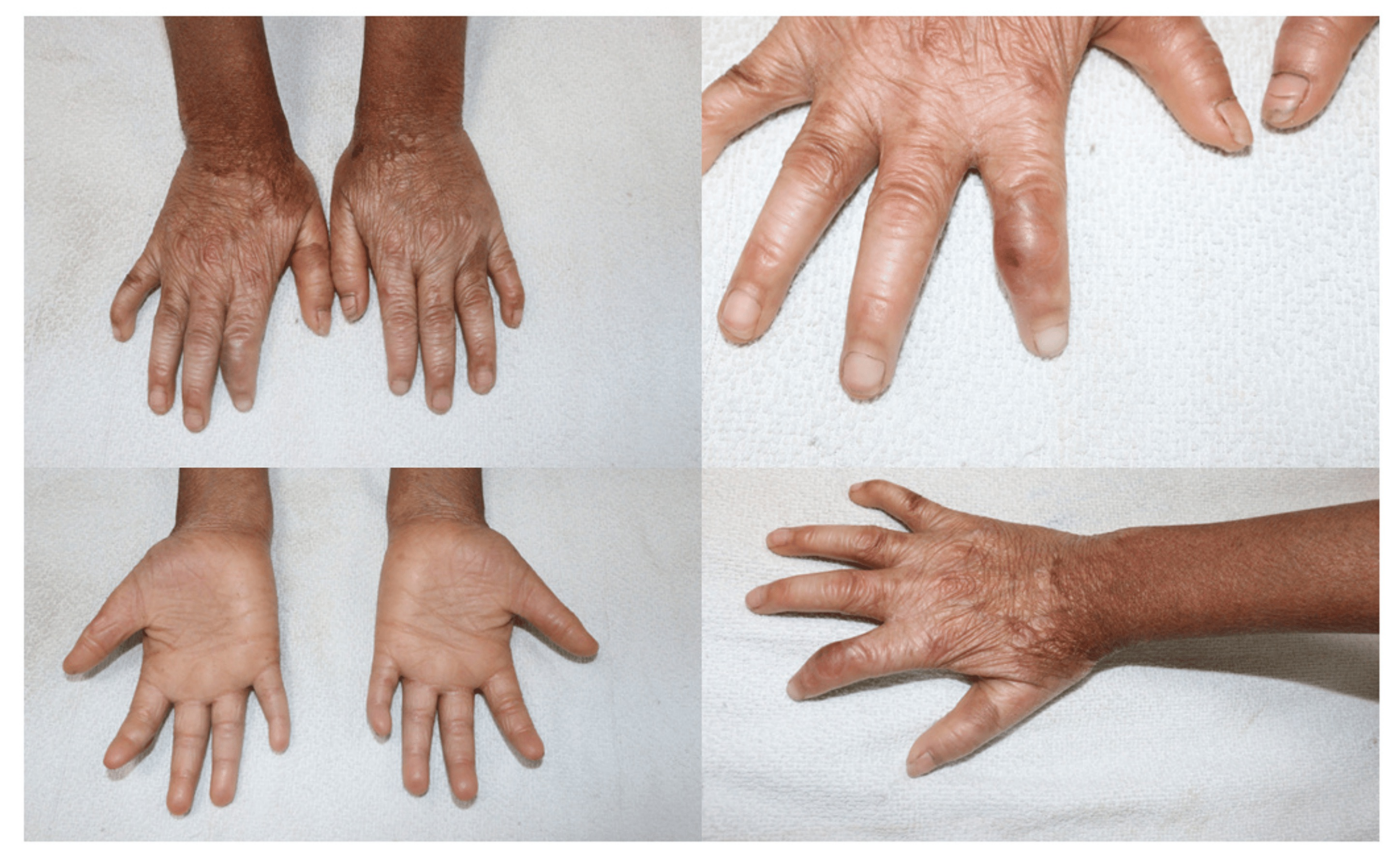
Extraoral manifestations on the upper limbs showing multiple trauma-induced bullae involving the fingers.

The hands also show similar involvement with xerosis, accentuated skin creases, and hyperpigmentation on the dorsum of both hands. One index finger of the right hand displayed a swollen and violaceous appearance, suggestive of a healing blister or subepidermal trauma. Nail plates of the hands were relatively preserved as compared to toes, but they also appeared mildly dystrophic (Figure 3).

Intraoral Examination

**Figure 4:**
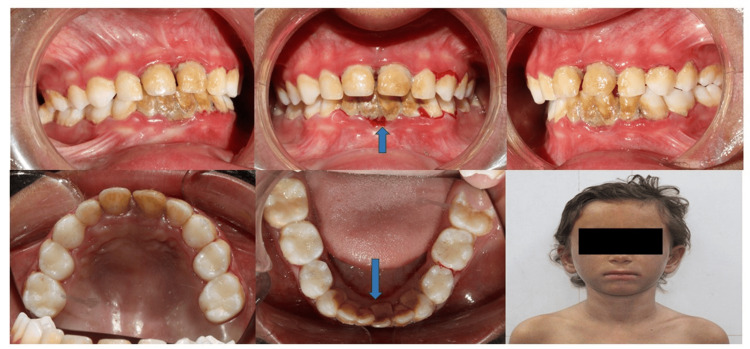
Pre-treatment intraoral profile displaying heavy lingual calculus on the lower anteriors causing profuse bleeding along with poor oral hygiene manifested by the presence of visible bleeding from the gums of the primary teeth.

After appropriate behavior management (tell, show, do, and voice control), she allowed for an intraoral checkup where, after the oral evaluation, she was found to have severe halitosis (based on organoleptic assessment) and severe accumulation of calculus over her lower mandibular anterior region spanning from lower left first primary molar (74) to lower right primary canine (83) lingually. Her poor oral hygiene practice has led to such heavy calculus accumulation. Profuse bleeding was seen from the distal surface of the lower left first primary molar (74) and also from the gingival crevices of the lower and upper anteriors labially on evaluation of gingival health (Figure 4), likely suggestive of plaque-induced gingivitis.

Clinical and oral management

The parents were advised by their respective dermatologists to wear the child loose clothes. They were instructed to avoid rigorous massage for the child and to take all the necessary precautions to prevent further trauma. They were prescribed a combination product containing 0.1% mometasone (w/w) and 2% fusidic acid (w/w) topical antibiotic cream to be applied three times on all the wounds to facilitate healing and reduce redness, swelling, and itchiness. An additional Kojitin© emulgel was also advised to reduce hyperpigmentation and provide protection against sunlight.

**Figure 5:**
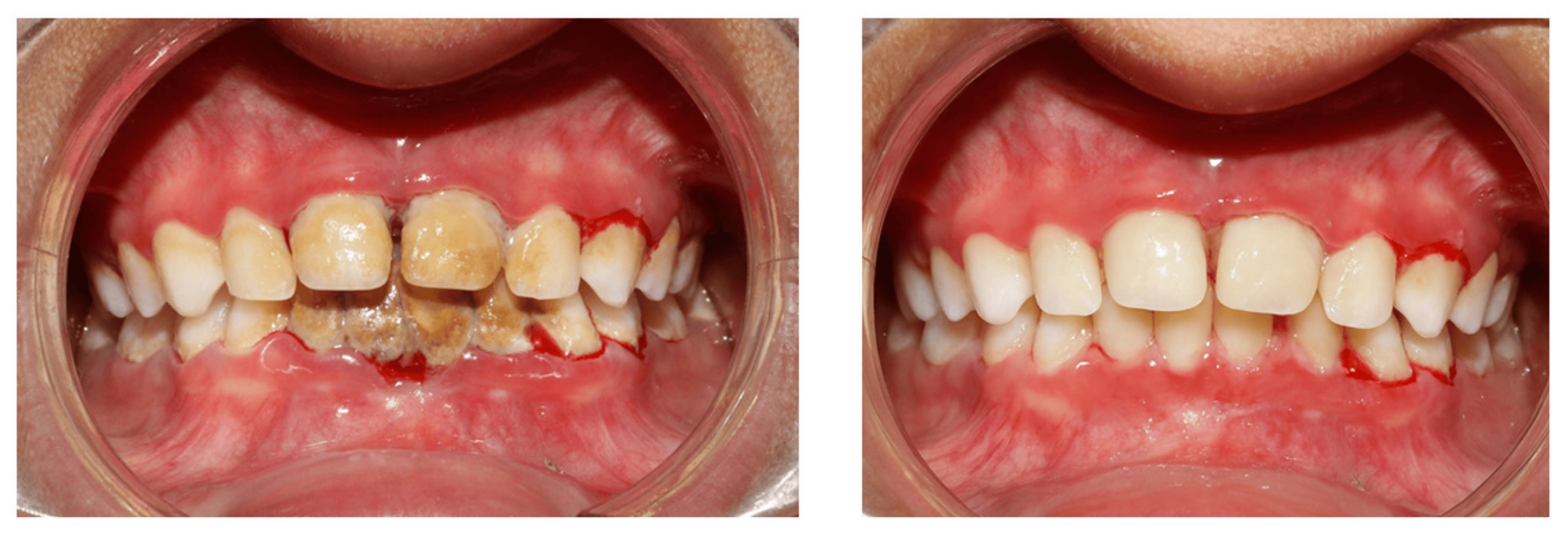
Post-treatment display of the intraoral profile after appropriate management of poor gingival health through scaling without causing any inadvertent trauma to the friable intraoral mucosa.

For oral care, after achieving adequate behavior management, the child was taken to the dental operatory for thorough scaling and removal of accumulated calculus deposits. Taking meticulous precautions, such as avoidance of mucosal contact, hand instruments were used initially, and later complete scaling was done with the help of an ultrasonic scaler (with reduced speed of water spray) in interdental areas to achieve complete removal of stains and deposits (Figure 5). The child, however, became uncooperative in between, which was appropriately managed using the voice control technique. The child was advised 1% sodium bicarbonate mouthwash to be swished three times daily for about one to two minutes for two weeks after a thorough discussion with her dermatologists.

## Discussion

EBS is a rare disease with multi-systemic involvement and thus requires a multidisciplinary care and management [7]. Although there is no gender prevalence proven as such for this condition, a female predilection has been reported in some studies [8]. Apart from dental problems, patients with generalized EB also suffer from persistent skin infections mostly due to *Staphylococcus aureus*, gastrointestinal problems such as esophageal dilatations, constipations, and celiac disease, poor growth manifested as growth delays and malnutrition, persistent anemia and nutritional deficiency, nephrological complications such as phimosis and renal failure, and endocrinological-thyroiditis, osteoporosis, hypothyroidism, and cardiac complications-rhythm alterations, inter-atrial defects, intra-ventricular defect, and acute myocardial infarctions depending upon the severity of EB [7]. Management of these types of genodermatoses requires inter-department coordination from all the specialities of dentistry, with pediatric dentists playing a central role in the management by providing appropriate awareness and dental education to the parents on oral hygiene, preventive strategies to improvise oral care, as well as addressing the current dental problems, such as restorative needs in primary, mixed, and permanent dentitions [9]. Another major factor to be considered is the prematurity and low birth weight (natal history) as seen in the patient presenting in the current case. Although the patient was born prematurely at 28 weeks with a low birth weight of 2 kg, no established association exists between prematurity and the development of EBS, which is a genetically determined disorder. However, certain rare variants, such as EB associated with pyloric atresia (EB-PA) and recessive dystrophic EB (RDEB), have been reported in preterm or low-birth-weight infants [10,11]. To the best of our knowledge, based on the available literature, this appears to be among the very few reported cases of clinically diagnosed EBS presenting in a prematurely born low birth weight child without associated pyloric atresia. In the present case, prematurity did not appear to influence the dermatological severity or oral manifestations observed.

Dental management in EB should adhere to a risk-based referral and follow-up protocol customized according to the clinical subtype and severity of the condition. Patients presenting with high-risk variants, such as junctional EB, generalized recessive dystrophic EB (RDEB), RDEB inversa, and Kindler EB, should be referred to a pediatric dentist at an early stage, ideally between three and six months of age, even before the tooth eruption. This should be complemented by vigilant monitoring throughout childhood and adolescence. Moderate-risk subtypes, including severe EB simplex, dominant dystrophic EB (DDEB), and localized RDEB, require dental assessment at the onset of initial tooth eruption around six months of age and then subsequent regular monitoring every six months. Conversely, standard dental care schedules that apply to the general population may be used to treat those with localized or intermediate EB simplex [12]. Oral manifestations in these patients vary depending on the severity and subtype of the disease, with involvement of soft and hard dental tissues. The majority of these patients exhibit vesiculobullous oral lesions of some kind, ranging from small, distinct vesicles to massive bullae and granulation tissue patches [13]. Generalised enamel hypoplasia is a common presentation in JEB as compared with other types. An increased caries experience is seen in patients with RDEB, whereas patients with Kindler EB have greater periodontal disease [13,14]. Although extensive plaque deposition has been reported in the majority of patients with RDEB, as seen in the current case, other manifestations of RDEB, such as the presence of serious bullae, the absence of palatal rugae, and depapillation on the tongue dorsum, were missing in our case [15,16]. However, genetic investigation of this case is awaited. All forms of EB require daily wound care, which includes granulation tissue management, bandage application and removal, lancing bullae, debriding crusts, administering emollients and antimicrobials, and wound assessment [17].

## Conclusions

This case highlights the importance of early dental referral, preventive-focused care, and atraumatic management strategies in pediatric patients with clinically diagnosed EBS. Timely multidisciplinary coordination is essential to prevent avoidable oral morbidity in children affected by this subtype. Genetic analysis would further strengthen subtype confirmation and remains a limitation of the present report.

Pediatric dentists play a central role in the multidisciplinary management of children with EBS. An early referral, preventive-focused protocols, gentle clinical handling, and continuous parental counselling are essential to maintain optimum oral health and improve the quality of life of children living with EB. Preventive Dentistry must remain the cornerstone of long-term care in these medically vulnerable patients.
